# Comparative Study on Grape Berry Anthocyanins of Various Teinturier Varieties

**DOI:** 10.3390/foods11223668

**Published:** 2022-11-16

**Authors:** László Kőrösi, Szilárd Molnár, Péter Teszlák, Ágnes Dörnyei, Erika Maul, Reinhard Töpfer, Tamás Marosvölgyi, Éva Szabó, Franco Röckel

**Affiliations:** 1 Research Institute for Viticulture and Oenology, University of Pécs, 7634 Pécs, Hungary; 2 Department of Analytical and Environmental Chemistry, Faculty of Sciences, University of Pécs, 7624 Pécs, Hungary; 3 Julius Kühn Institute (JKI)—Federal Research Centre for Cultivated Plants, Institute for Grapevine Breeding Geilweilerhof, 76833 Siebeldingen, Germany; 4 Institute of Bioanalysis, Medical School, University of Pécs, 7624 Pécs, Hungary; 5 Department of Biochemistry and Medical Chemistry, Medical School, University of Pécs, 7624 Pécs, Hungary

**Keywords:** bioavailability, dyer grape, genetic fingerprinting, HPLC, Orbitrap LC-MS, red-fleshed grape, SSR marker, *Vitis rupestris*, *Vitis vinifera*, *VvmybA1*

## Abstract

The red-fleshed grape cultivars, called teinturier or dyer grapes, contain anthocyanins in both the skin and flesh. These phenolic compounds exhibit excellent coloring ability, and as antioxidants, they are important bioactive compounds in food crops. In this work, anthocyanin patterns of grape berries of fifteen teinturier varieties collected from the gene bank located at Pécs in the southwest of Hungary were compared. Anthocyanin profiles of numerous varieties originating from Hungary such as ‘Bíborkadarka’, ‘Kármin’, ‘Kurucvér’, and ‘Turán’ are reported for the first time. Anthocyanins extracted separately from the skin and juice were analyzed using high-performance liquid chromatography coupled with a photodiode array detector. For the identification of compounds, high-resolution orbitrap mass spectrometry was used. All in all, twenty-one anthocyanins were identified and quantified. We found that anthocyanin patterns differed significantly in the skin and juice for all investigated cultivars. For *Vitis vinifera* varieties, the predominant anthocyanin in the skin was malvidin-3-*O*-glucoside, while the main pigment in the juice was peonidin-3-*O*-glucoside. For the first time, a significant amount of diglucosides was detected in two *Vitis Vinifera* cultivars with a direct relationship. In general, the pigment composition of the skin was much more complex than that of the juice. The comparative study with presented patterns gives valuable and beneficial information from a chemotaxonomical point of view. Our results also help to choose the appropriate teinturier varieties with the desired anthocyanins for food coloring or winemaking purposes.

## 1. Introduction

**Figure 1:**
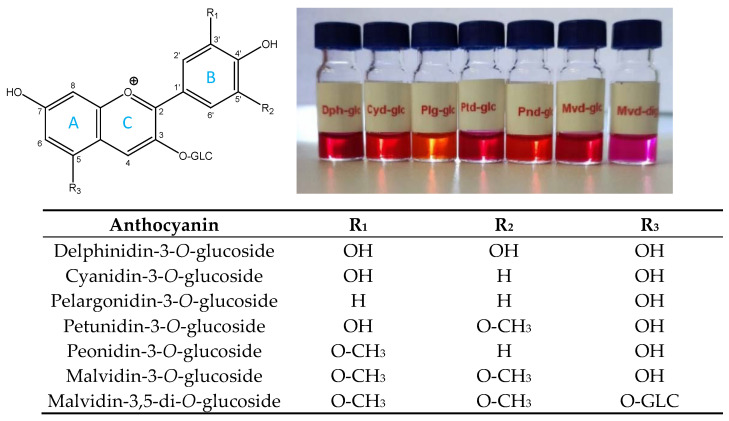
The major anthocyanins of *Vitis* species: structure of the flavylium cation substituted with OH, O−CH_3_, and *O*-glucoside. For acylated anthocyanins, acetyl- or coumaroyl-glucoside substitution occurs at C_3_ position on C ring. The image demonstrates the color of the anthocyanins solved in methanol acidified with 0.1% HCl. From left to right: Delphinidin-3-*O*-glucoside (Dph-glc), Cyanidin-3-*O*-glucoside (Cyd-glc), Pelargonidin-3-*O*-glucoside (Plg-glc), Petunidin-3-*O*-glucoside (Ptd-glc), Peonidin-3-*O*-glucoside (Pnd-glc), Malvidin-3-*O*-glucoside (Mvd-glc), and Malvidin-3,5-di-*O*-glucoside (Mvd-diglc).

Teinturier grapes (also called dyer or red-fleshed grapes) occupy a special place among grapevine varieties. Both their skin and pulp contain various anthocyanins as red pigments [1]. The general formula of the anthocyanins in grapes is shown in Figure 1. Their chemical structure comprises a C_6_-C_3_-C_6_ flavan (2-phenyl-benzodihydropyran) skeleton, which can be split into two aromatic rings (denoted A and B) and a heterocyclic ring with three carbon atoms (ring C) as represented in Figure 1. Anthocyanins, therefore, belong to the subgroup of flavonoids. The flavylium cation can be substituted in positions 3, 5, 7, 3′, 4′, and 5′, depending on the compound. The substituents are hydroxyl or methoxy and glycosyl groups (Figure 1).

The sugar moiety is glucose (glucoside) and/or acetyl- or cinnamoyl derivatives of glucose (acetylated- or coumarylated glucosides) [2,3]. The glucose is linked to the skeleton by a glycosyl bond at position 3 of the C ring (so-called monoglucosides). For diglucosides, glycosidic bonds are found both in position 3 of the C ring and position 5 of the A ring. The part of the molecule without sugar moiety is called anthocyanidin aglycone. The glycosylation of anthocyanidins in plants is very important, affecting the physicochemical properties of the molecule (e.g., stability and hydrophilicity or water solubility). The structure of anthocyanins is pH-dependent, which basically determines the color of the chromophore. In an acidic medium, the flavilium cation is red (Figure 1), whereas in a basic medium, the quinoidal form is blue. In addition, colorless carbinol (pseudobase) and yellow chalcone forms are also known [4,5]. The acidic pH in the berries is provided by the organic acids such as tartaric acid, malic acid, and citric acid.

Because of their excellent coloring ability, the wines made from teinturier berries are of great importance in wine-making technology for blending to enhance the color of red wines. In addition to coloring wine, these anthocyanins can also be used as food colors with several advantages. First of all, they have been consumed since ancient times without adverse effects on health; furthermore, they are water-soluble compounds, allowing incorporation into aqueous food systems [6]. Anthocyanins belong to the subclass of phenolic phytochemicals and are widespread plant pigments in nature [7]. The composition or pattern of anthocyanins is very characteristic of a given plant species, and consequently, they are very important markers from a chemotaxonomic point of view. In addition to the aforementioned food–chemical benefits, the red pigments in teinturier grapes can also play an important role in human nutrition [7,8,9,10]. As dietary supplements, anthocyanin-containing foods may play a role in the prevention or complementary treatment of many diseases, such as reducing the risk of certain types of cancer [10,11,12]; reducing blood cholesterol, triacylglycerol and low-density lipoprotein [9,12,13,14,15], and lipid oxidation [16,17,18,19,20]; and decreasing fasting blood glucose and glycated hemoglobin [9,14,15,19,21,22], systemic and vascular inflammation [9,12,23], and body mass index and body weight [14,17,21,22,24], especially in long-term use. Furthermore, anthocyanins show antimicrobial activity against foodborne pathogens [20,25] and can promote the proliferation of beneficial *Bifidobacteria* while inhibiting pathogens, such as *Clostridium* in in vitro studies [26].

The spread of teinturier grapes dates back to the first half of the 19th century when Louis Bouschet created the variety ‘Bouschet Petit’ by crossing ‘Aramon’ and teinturier varieties. Henry Bouschet crossed ‘Bouschet Petit’ with ‘Grenache Noir’ to produce ‘Alicante Bouschet’, which has become one of the most cultivated varieties in northwest Spain [1]. In the second half of the 19th century, a number of new varieties such as ‘Terret Bouschet’ (‘Terret’ × ‘Bouschet Petit’) and ‘Muscat Bouschet’ (‘Muscat Fleur D’oranger’ × ‘Bouschet Petit’) were created by breeding. The spread of teinturier cultivars in the early 20th century was motivated not only by the production of deeper-colored wines but also by the downy mildew (*Plasmopara viticola*) [27] and powdery mildew (*Uncinula necator*), which then emerged in Europe [1]. The emergence of these grape pathogens was also triggered by the breeding of new, more resistant but still good wine-quality grape varieties, including teinturier ones. North American varieties were also included in their breeding to increase resistance. The gene bank of the Research Institute for Viticulture and Oenology at the University of Pécs has a number of teinturier varieties. Hungarian varieties include ‘Bíborkadarka’, ‘Kármin’, ‘Kurucvér’, and ‘Turán’. Their pedigrees have not been confirmed by markers yet, and there is little or no international literature data on their anthocyanin profiles. Indeed, only a few articles are available focusing on anthocyanins of teinturier grapes. For instance, Castillo-Muñoz et al. [28] studied the phenolic composition of ‘Garnacha Tintorera’ (also known as ‘Alicante Henri Bouschet’), and they found that anthocyanins were asymmetrically distributed between the grape flesh and skins. The anthocyanins of ‘Yan73’ (‘Muscat Hamburg’×‘Alicante Henri Bouschet’) were reported by He et al. [29]. Guan et al. [30] showed that light exclusion reduced the concentration and modified the composition of grape anthocyanins in ‘Gamay Teinturier Fréaux’. The analysis of anthocyanin biosynthesis at the genetic and molecular level in red-fleshed grape berries was studied by two research groups [8,31], and recently, a multiple 408 bp repeat in the promoter of *VvmybA1* (respective allele called *MybA1t*) could be associated with the typical phenotype of red berry flesh and foliage of the well-known teinturier grapevine varieties [32]. Both the white (non-functional; *MybA1a*) and red (functional; *MybA1c*) alleles contain just a single copy of the repeated fragment and lead to non-colored and colored berry skins, respectively, with colorless flesh.

Although teinturier grapes are important and valuable fruits because of their high anthocyanin content, no comprehensive comparative study including a large number of teinturier varieties has been reported yet. Therefore, our aim was to describe and compare the anthocyanin profiles of various teinturier grapes including a famous European variety (*Vitis vinifera*) and an interspecific cross. Besides qualitative analysis, the quantification of the individual anthocyanins for each genotype was also performed.

## 2. Materials and Methods

### 2.1. Chemicals and Reagents

Acetonitrile and methanol (Promochem Optigrade, LGC Standards GmbH, Wesel, Germany) were gradient grades for liquid chromatography. Delphinidin-3-*O*-glucoside (Dph-glc), Cyanidin-3-*O*-glucoside (Cyd-glc), Pelargonidin-3-*O*-glucoside (Plg-glc), Petunidin-3-*O*-glucoside (Ptd-glc), Malvidin-3,5-di-*O*-glucoside (Mvd-diglc), Peonidin-3-*O*-glucoside (Pnd-glc), and Malvidin-3-*O*-glucoside (Mvd-glc) were purchased from Extrasynthese (Genay, France). Formic acid 98–100% was purchased from Molar Chemicals Ltd., Halásztelek, Hungary. High-purity deionized water was obtained by a LaboStar 7 TWF-UV ultrapure water system (SG Wasseraufbereitung und Regenerierstation GmbH, Barsbüttel, Germany).

### 2.2. Experimental Site and Plant Materials

For the investigations, the berries of different teinturier grapevines were collected from the gene bank of the University of Pécs on the south-facing slopes of Mecsek Hills in Hungary (latitude: 46°04′ N, longitude: 18°11′ E, 150 m a.s.l.). Grapevine varieties used in this study are listed in Table S1 in the Supplementary Materials. Vines were grafted on commonly used rootstock varieties ’T5C’ (*Vitis berlandieri* × *Vitis riparia*) and grown under non-irrigated open-field conditions. They were planted in a north–south row direction with 2 (between the rows) ×1 m (within the row) vine spacing using a mid-high cordon trellis system. The soil was a Ramann-type brown forest soil mixed with clay formed on red sandstone covered by Pannonian sediment. Meteorological data were monitored using the WS600 automatic weather station (OTT HydroMet Fellbach GmbH, Fellbach, Germany). In 2020, the average annual temperature was 19.4 °C, and the site received 471 mm of annual precipitation and 2067 h of sunshine. Grape berries were harvested between September and October 2020 at optimal technological ripeness. One hundred berries were collected from ten randomly selected clusters and five trunks. The harvesting parameters including the average mass of whole berries, pH, total solid content (TSS), and acid profile of juice samples are summarized in Table S2. TSS measurements were performed using Atago Digital Wine Refractometer—WM-7 (ATAGO Co., Ltd., Tokyo, Japan) and expressed in °Brix. For DNA analysis, leaf samples were collected in liquid nitrogen and then subsequently freeze-dried for 24 h using a ScanVac CoolSafe 110-4 Freeze Dryer (LaboGene ApS, Allerod, Denmark).

### 2.3. Genetic Fingerprinting and Determination of the MybA1t Repeat Number at the Berry Color Locus (BCL)

DNA was extracted from young leaves with the Plant DNA Mini Kit (Peqlab, Erlangen, Germany) following the supplier’s instructions. Samples were genotyped with nine genome-wide simple sequence repeat (SSR) markers to confirm trueness-to-type identity [33] according to Huber et al. [34]. The MybA1t repeat number as well as the MybA1a and MybA1c allele status at the BCL of the analyzed varieties was determined as described previously by Röckel et al. [32]. A complete variety list with the genetic profiles and BCL allele status is shown in Table S1.

### 2.4. Analysis of Organic Acids

The main organic acids of juice samples were analyzed on a Shimadzu Prominence UFLC system (Shimadzu Co., Kyoto, Japan) using RID-10A refractive index detector. Chromatographic separation of tartaric-, malic-, and citric acid was achieved on a Phenomenex Synergy 4 µm Hydro-RP 80 Å, 250 × 4.6 mm LC column. Isocratic elution was performed using the eluent of 20 mM KH_2_PO_4_ in 150 mM H_3_PO_4_, with a flow rate of 1 mL/min. The column temperature was maintained at 45 °C. The volume of the injected sample was 10 µL.

### 2.5. Extraction of Berries’ Skin

One hundred berries in the frozen state were peeled, and the resulting skins were crushed into a fine powder in liquid nitrogen. A total of 5 mg of each skin sample was sonicated in 1 ml of methanol acidified with 0.1% HCl for 30 min using an Elma Transsonic T 460 ultrasonic bath with a noise frequency of 35 kHz (Singen/Hohentwiel, Germany). The resulting suspension was centrifuged at 20,660× *g* for 10 min, and subsequently, the extraction of the sediment was repeated. The obtained supernatants were merged, and then the solvent was evaporated by using N_2_ gas and a block heater (Grant QBD4 Digital Dry Block Heater, Grant Instruments (Cambridge) Ltd., Sepreth, UK). Dried residues were redispersed in 500 µL of 50% methanol and filtered through a 0.22 µm PES syringe filter (FilterBio^®^, Labex Ltd., Budapest, Hungary) before HPLC analysis.

### 2.6. Preparation of Juice Samples

After removing the skin part from the frozen berries, the berry flesh was allowed to defrost at room temperature, and then they were gently pressed to obtain juice. Prior to HPLC analysis, the juice samples were centrifuged at 20,660× *g* for 10 min and finally filtered through a 0.22 µm PES syringe filter (FilterBio^®^, Labex Ltd., Budapest, Hungary).

### 2.7. Analysis of Anthocyanins

HPLC-DAD analysis was performed on a Shimadzu Prominence UFLC system (Shimadzu Co., Kyoto, Japan) consisting of an online degassing unit (DGU-20A5R), pump (LC-20AD), column oven (CTO-20AC), autosampler (SIL-20AC HC), diode array detector (SPD-M20A), and fraction collector (FRC-10A). The chromatographic separation was conducted on a Kinetex^®^ 2.6 µm Polar C18 100 Å, 100 × 4.6 mm LC Column. The column temperature was kept at 40 °C. Gradient elution was applied using 10% (*v*/*v*) formic acid (A) and a mixture of acetonitrile (90% *v*/*v*) and formic acid (10 % *v*/*v*) (B). Gradient elution started at 97% (*v*/*v*) A, ramping up to 21% B over 37 min. The flow rate of the mobile phase was kept at 1.0 mL/min; the volume of the injected sample was 5 μL. For the evaluation, the absorbance values acquired at 530 nm were used. Calibration curves were obtained by measuring commercially available anthocyanins with known concentrations. For commercially unavailable anthocyanins, semiquantitative analysis was performed using Mvd-glc as a reference compound. The results were expressed in µg anthocyanin per mg fresh weight (FW) for berry skin, or in mg/L for juice.

All the chromatographically separated components were collected by Shimadzu Fraction collector (FRC-10A). The fractions collected were analyzed by a Thermo Q Exactive Focus hybrid Quadrupole-Orbitrap mass spectrometer (Thermo Fisher Scientific, Waltham, MA, USA). Samples were acquired in Full Scan and All Ion Fragmentation modes in a range from 100 to 1000 m/z. The ionization was carried out via a heated electrospray ionization (HESI) source run in positive and negative switched modes. The operating parameters are summarized in Table 1. Data analysis and evaluations were performed using the Q Exactive Focus 2.1 and Xcalibur 4.2. software (Thermo Fisher Scientific, Waltham, MA, USA).

### 2.8. Statistical Analysis

Statistical analysis was performed using IBM SPSS 28.0 Statistics for Windows (SPSS Inc., Chicago, IL, USA). The results were statistically analyzed by calculating the mean and standard deviation. Spearman’s rank correlation was applied to analyze the relationships between the concentration of each anthocyanin in grape skin, juice, and between skin and juice samples. Hierarchical cluster analysis of the anthocyanin content of both the grape skin and juice was performed using Ward linkage and squared-mean Euclidean distance.

## 3. Results and Discussion

**Figure 2:**
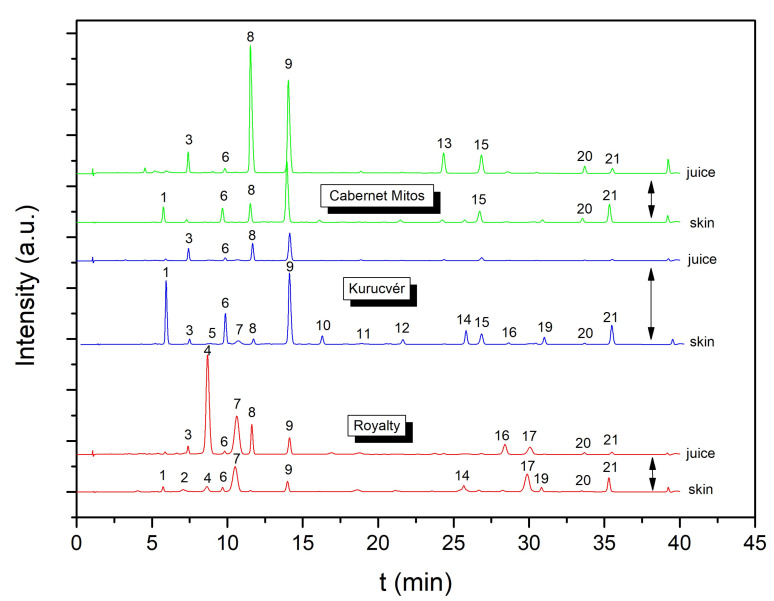
HPLC-DAD chromatograms of skin extract and juice for ‘Royalty’, ‘Kurucvér’, and ‘Cabernet Mitos’ grape varieties. Identified compounds designated by numbers are summarized in Table 2.

High-performance liquid chromatographic analysis was performed in order to describe and compare the anthocyanin profile of various teinturier grapevine cultivars. In this study, we focused on *Vitis vinifera* varieties, but one interspecific hybrid was also investigated. For all the studied varieties, trueness-to-type identity was confirmed by genetic fingerprinting (Table S1). To illustrate their diverse anthocyanin compositions in both skin and juice, some typical HPLC-DAD chromatograms are demonstrated in Figure 2. Twenty-one well-separated anthocyanins were identified. These compounds, with their characteristic retention times and the mass of their molecular and fragment ions, are summarized in Table 2. The results showed that anthocyanins of the presented teinturier varieties are the different glucosides of delphinidin, cyanidin, pelargonidin (with trace level), petunidin, peonidin, and malvidin. These anthocyanins, typical of *Vitis* species, are among the simplest in higher plants [35].

**Figure 3:**
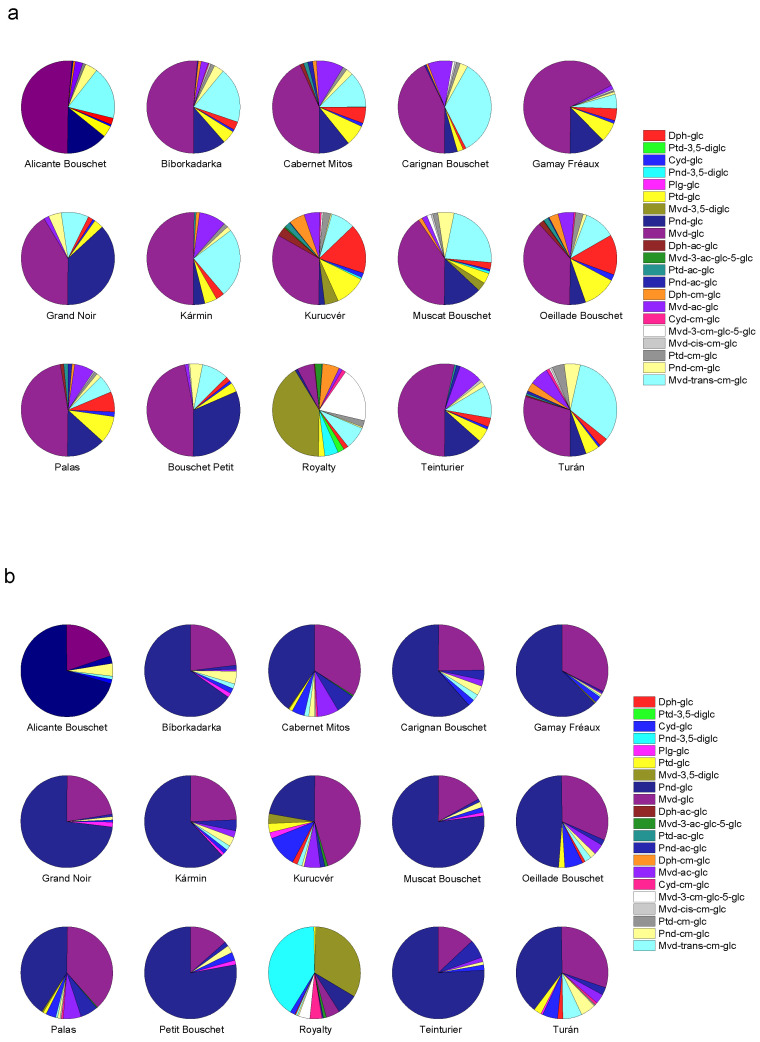
Anthocyanin profiles of (**a**) skin and (**b**) juice of various teinturier grapevine cultivars. Dph-glc: Delphinidin-3-*O*-glucoside, Ptd-3,5-diglc: Petunidin-3,5-di-*O*-glucoside, Cyd-glc: Cyanidin-3-*O*-glucoside, Pnd-3,5-diglc: Peonidin-3,5-di-*O*-glucoside, Plg-glc: Pelargonidin-3-*O*-glucoside, Ptd-glc: Petunidin-3-*O*-glucoside, Mvd-3,5-diglc: Malvidin-3,5-di-*O*-glucoside, Pnd-glc: Peonidin-3-*O*-glucoside, Mvd-glc: Malvidin-3-*O*-glucoside, Dph-ac-glc: Delphinidin-3-*O*-(6-*O*-acetyl-glucoside), Mvd-3-ac-glc-5-glc: Malvidin-3-O-(6-*O*-acetyl-glucoside)-5-*O*-glucoside, Ptd-ac-glc: Petunidin-3-*O*-(6-*O*-acetyl-glucoside), Pnd-ac-glc: Peonidin-3-*O*-(6-*O*-acetyl-glucoside), Dph-cm-glc: Delphinidin-3-*O*-(6-*O*-p-coumaroyl-glucoside), Mvd-ac-glc: Malvidin-3-*O*-(6-*O*-acetyl-glucoside), Cyd-cm-glc: Cyanidin-3-*O*-(6-*O*-p-coumaroyl-glucoside), Mvd-3-cm-glc-5-glc: Malvidin-3-*O*-(6-*O*-coumaroyl-glucoside)-5-*O*-glucoside, Mvd-cis-cm-glc: Malvidin-3-*O*-(6-*O*-cis-p-coumaroyl-glucoside), Ptd-cm-glc: Petunidin-3-*O*-(6-*O*-trans-p-coumaroyl-glucoside), Pnd-cm-glc: Peonidin-3-*O*-(6-*O*-trans-p-coumaroyl-glucoside), Mvd-trans-cm-glc: Malvidin-3-*O*-(6-*O*-trans-p-coumaroyl-glucoside).

The compositions of skin and juice samples for the main anthocyanins are listed in Table 3, while the semiquantitative results with derivative compounds are reported in Tables S3 and S4. The anthocyanin patterns of different cultivars are visualized using pie charts in Figure 3a,b. The obtained results showed that anthocyanin composition is significantly different in the skin and juice, even in the same genotype. This finding is in agreement with previous results. The varieties ‘Kolor‘ and ‘Yan73′, for instance, similarly exhibit different anthocyanin profiles within the berries [29,36,37,38,39]. Based on our measurements, while the skins contained many kinds of anthocyanins, the juices’ composition was simpler, showing a higher abundance of Pnd-glc and Mvd-glc. In most cases, the ratio of these compounds was ~70–95% in the juice, while it was below 70% in the skin.

For all the cultivars with the exception of ‘Royalty’, Mvd-glc was the most abundant anthocyanin with a level of 3894–19,102 µg/g FW in the skin (Table 3). For five cultivars, namely, ‘Alicante Bouschet’, ‘Bíborkadarka’, ‘Gamay Teinturier Fréaux’, ‘Kármin’, and ‘Teinturier’, the amount of malvidin-3-*O*-glucoside was >50% of the total anthocyanin content (Figure 3a). Berries’ skin of ‘Royalty’ contained the lowest level of Mvd-glc (2207 µg/g FW; 6.0%). In this cultivar, Mvd-3,5-diglc (15,264 µg/g FW; 41.2%) and Mvd-3-ac-glc-5-glc (910 µg/g FW; 19.1%) were the major anthocyanins. For all the cultivars, a common malvidin derivative was the Mvd-trans-cm-glc. Its level varied in the range of 900–7240 µg/g FW. In some teinturier varieties, Pnd-glc was found to be significant. The berries’ skin of ‘Grand Noir’ and ‘Petit Bouschet’ contained Pnd-glc at a high ratio and relatively high concentration (3468–3959 µg/g FW). Dph-glc at a higher level was found in only two cultivars, namely, ‘Oeillade Bouschet’ (7030 µg/g FW) and ‘Kurucvér’ (7930 µg/g FW). These two cultivars had similarly high Ptd-glc levels (5890 µg/g FW and 4990 µg/g FW, respectively). In contrast, Cyd-glc was detected in almost all grape varieties. In the skin, it was again found at the highest concentration in ‘Oeillade Bouschet’.

In the juice samples, for most of the varieties, Pnd-glc was the predominant anthocyanin (Figure 3b). Its concentration varied between 42.4 and 381.6 mg/L (Table 3). Mvd-glc was dominant in the juice sample of ‘Kurucvér’. An also typical characteristic of the berry juice of ‘Kurucvér’ is its relatively high Cyd-glc ratio (11.1%). Other cultivars produced a significantly lower proportion of Cyd-glc (1.0–6.4%). The concentration of Plg-glc reached 2–4 mg/L in the berry juice for several varieties such as ‘Kurucvér’, ‘Bíborkadarka’, and ‘Grand Noir’. This anthocyanin is typically not or hardly detected in *Vitis vinifera* grape berries [40]. Like the skin, the composition of ‘Royalty’ berries’ juice was utterly different, containing two dominant anthocyanins, Pnd-3,5-diglc and Mvd-3,5-diglc, at very high concentrations (434.0 and 349.7 mg/L, respectively). Dph-glc was only detectable as trace in all but four varieties: the highest Dph-glc content was found in ‘Turán’, followed by ‘Kurucvér’, ‘Royalty’, and ‘Oeillade Bouschet’. In the former studies, Dph-glc was also found in relatively low amounts in ‘Yan 73’ [29,36,37,39], ‘Kolor’ [29,36], ‘Salt Greek’ [29], and ‘Gamay de Bouze’ and ‘Gamay Fréaux’ [41], but in relatively high amounts in ‘Yan 74’ (10.5 mg/g) [29] and ‘Summer Black’ (109.76 mg/100 g) and ‘Summer Black mutant’ (282.8 mg/100 g) [42].

Not all twenty-one anthocyanins were found in each cultivar. For example, both skin and juice samples of ‘Royalty’, which is an interspecific cross, contained relatively large amounts of diglucosides, namely, Ptd-3,5-diglc, Pnd-3,5-diglc, and Mvd-3,5-diglc (Table 3, Tables S3 and S4). In contrast, a significant amount of Mvd-3,5-diglc was not detected in *Vitis vinifera* species except for ‘Kurucvér’ and ‘Muscat Bouschet’ varieties. The chemotaxonomic significance of anthocyanins was revealed earlier [43]. Mvd-3,5-diglc is a characteristic anthocyanin for *Vitis rupestris* [29,44] as well as *Vitis amurensis* species [45,46]. *Vitis labrusca* also contains a high level of diglucosides [44,47]. While several non-vinifera species produce diglucosides, it is totally unexpected that ‘Muscat Bouschet’ and ‘Kurucvér’, two *Vitis vinifera* varieties, also contain Mvd-3,5-diglc. In the skin sample, 543 µg/g and 2168 µg/g was found for ‘Muscat Boushet’ and ‘Kurucvér’, respectively. This is the first report of such high diglucoside content for *Vitis vinifera* berry skins. ‘Kurucvér’ is the progeny of ‘Muscat Bouschet’ and Kadarka and could therefore inherit the trait by natural crossing from ‘Muscat Bouschet’. The direct relationship between ‘Kurucvér’ and ‘Muscat Bouschet’ was confirmed by nine SSR markers (Table S1). It should be noted that the pedigree as given by the breeder showed that ‘Bíborkadarka’ was created by crossing ‘Kadarka’ and ‘Muscat Bouschet’. However, the genetic profiles obtained did not confirm that ‘Bíborkadarka’ is the progeny of ‘Muscat Bouschet’. In line with this, ‘Bíborkadarka’ did not contain Mvd-3,5-diglc. The anthocyanin profile depends mainly on the grape genotype but can also be influenced by environmental parameters. For example, Xing et al. described an uncommon anthocyanin profile for Cabernet Sauvignon berries grown in a high-altitude region [48]. They found that the altitude of the vineyard had a significant effect on anthocyanin accumulation. Furthermore, higher cultivation altitude (approximately between 2150–2900 m a.s.l.) not only increased the anthocyanin level but three diglucosides (namely, Pnd-3,5-diglc, Dph-3,5-diglc, and Mvd-3,5-diglc) not typical of *V. vinifera* were detected in the berries. An unusual anthocyanin composition was also described for wines made from *V. vinifera* grapes, such as Cabernet Sauvignon, Shiraz, and Marselan, in which Dph-3,5-diglc and Mvd-3,5-diglc were detected [49]. In that study, Mvd-3,5-diglc was in the concentration range of 7.9–10.9 mg/L, which is unrealistically high in *V. vinifera* wines. However, in our case, its unusual presence in Muscat Bouschet berries cannot be explained by geographical conditions or environmental parameters. It is more likely that Mvd-3,5-diglc in this variety stems from a diglucoside-producing ancestor. It should be noted that the muscat cultivars are thought to be very old, and their origin/genetic background is still unclear (Figure S1). We are planning further research to clarify why these *Vitis vinifera* cultivars produce diglucosides at a high concentration.

Regarding the main anthocyanins in the skin, very strong positive correlations were found between the concentration of Dph-glc, Cyd-glc, Ptd-glc, and Mvd-glc (Table 4). In juice, also strong positive correlations were established between the concentration of Cyd-glc, Pnd-glc, and Mvd-glc (Table 4). The obtained heat map in Table 4 also shows that the relations between individual anthocyanins differed in skin and juice. In the comparison of skin and juice, significantly lower number of strong correlations were obtained in juice. In this case, only Cyd-glc and Ptd-glc as major anthocyanins were strongly correlated with each other. The biosynthesis of anthocyanins in plants occurs via the flavonoid pathway and is strictly controlled through the involvement of several regulatory genes [50,51]. The accumulation of anthocyanins is influenced by many factors such as genetic regulation, phytohormones, and various environmental factors. In grapevine, several R2R3-Myb transcriptional factors, for example the *VvmybA* genes (*VvmybA1*, *VvmybA2,* and *VvmybA3*), were identified, which take part in the regulation of the phenylpropanoid pathway. Furthermore, the *MybA1t* allele exclusively found in teinturier varieties suggests autoregulation of the *VvmybA1* expression, leading to enhanced coloration in berry skin and flesh [32]. The aforementioned significant positive correlations between some individual anthocyanins indicate that their biosynthetic regulation is similar. Nevertheless, since the correlations between the compounds are different in skin and juice for the same variety, we can also conclude that these regulations are tissue-specific. These results are in good agreement with previous studies describing a tissue-specific expression of *VvF3’H*, *VvF3’5’H1*, and *VvCytoB5*, which play a key role in flavonoid synthesis and accumulation in grapes [52].

Based on the anthocyanin composition of the grape skin and juice samples, a number of similarities were found among the grape varieties on the dendrograms, with good agreement with their pedigree-based relationship (Figures S1 and S2). Descendants of ‘Petit Bouschet’ showed a very high statistical similarity in the anthocyanin composition. ‘Royalty’, the only interspecific cross in the dataset (genetic background most likely *Vitis rupestris*), was clearly distinct from the other cultivars, forming a separate group for both skin and juice samples. ‘Palas’ and ‘Cabernet Mitos’ showed very high similarities in the juice composition. Additionally, ‘Oeillade Bouchet’ and ‘Kurucvér’ were found in the same group as shown in the dendrogram of the skin samples. The dendrograms associated with the skin and juice samples showed different relationships between the varieties, underlying their different anthocyanin composition. Furthermore, the higher simplicity of the juice composition makes it difficult to distinguish between varieties. Nevertheless, anthocyanin composition can be useful for tracing the relationship between the grape varieties and for chemotaxonomic classification. Previous studies have also found similarities in anthocyanin [43,53,54] or phenolic content [55,56] in grapes as well as in wines made from the same grape variety [57,58].

**Figure 4:**
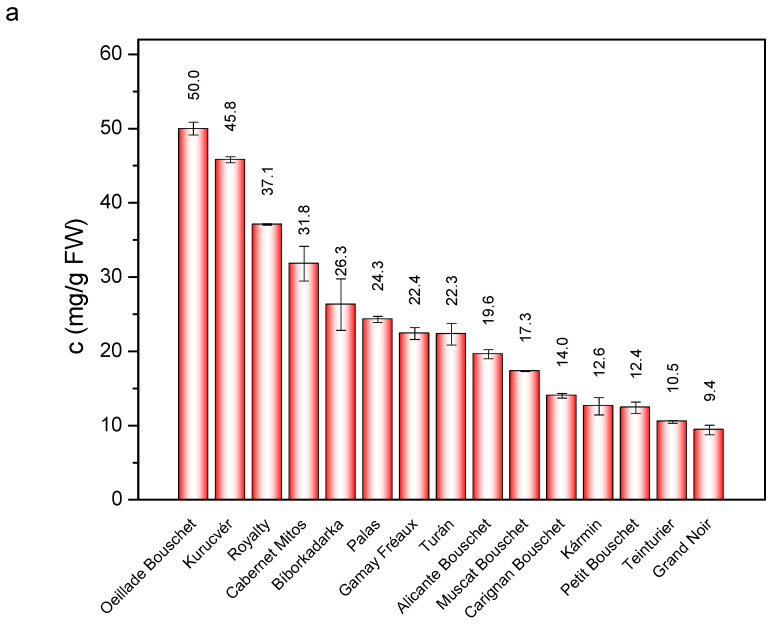

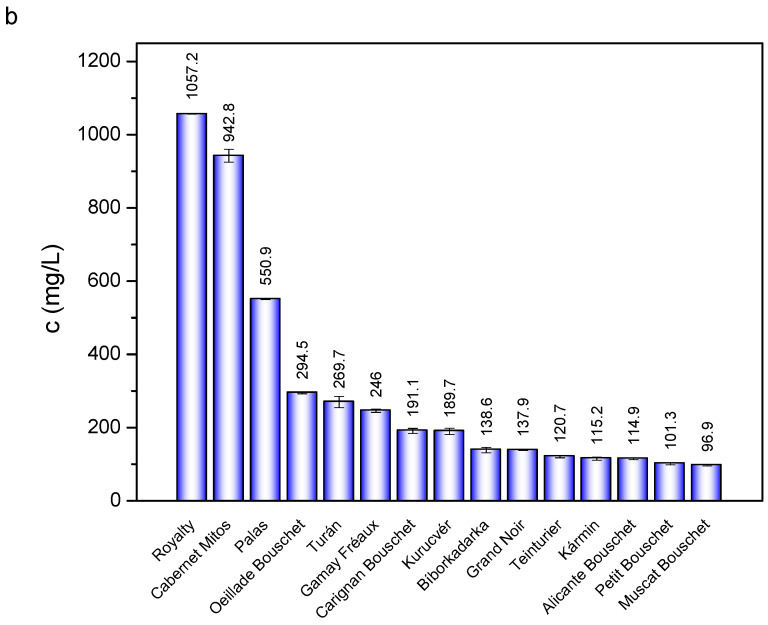
Total anthocyanin contents in (**a**) skin and (**b**) juice samples. The data are expressed in mg/g fresh weight (FW) and mg/L for skins and juices, respectively.

There were significant differences between the investigated varieties not only in anthocyanin composition but also in total anthocyanin content, which was calculated by summarizing the individual anthocyanin levels obtained by HPLC-DAD analysis. The results are shown in Figure 4. For berry skin, ‘Oeillade Bouschet’ and ‘Kurucvér’ contained the highest level of anthocyanins (50.0 and 45.8 mg/g FW), while ‘Royalty’ and ‘Cabernet Mitios’ was the richest anthocyanin source for the berry juice (1057.2 and 942.8 mg/L). The anthocyanin content is important not only for coloring ability but also for nutritional and therapeutic purposes. They rapidly absorb into the body and are able to cross the blood–brain barrier, thus exerting a neuroprotective effect by direct scavenging of ROS [59]. Furthermore, by modulating the enzymatic defense system, they can upregulate the activity of various enzymes such as SOD, CAT, GPx, etc. [60]. Because of their high anthocyanin content, the aforementioned four grape varieties are therefore of particular importance. To sum up, teinturier grapes are excellent sources of anthocyanins.

## 4. Conclusions

Grape berries of teinturier varieties are very rich in anthocyanins. These pigments accumulate mainly in the skin of the berries, and their total levels can reach ~50 mg/g FW for ‘Oeillade Bouschet’ and ‘Kurucvér’ varieties. The anthocyanin levels in the berry juice are also highly dependent on the genotype. For two varieties, ‘Royalty’ and ‘Cabernet Mitos’, their concentration was around 1000 mg/L. Moreover, we found that anthocyanin composition differed significantly in the skin and juice for each cultivar. Prevailingly, for *Vitis vinifera* varieties, the predominant component was malvidin-3-*O*-glucoside in the skin, while the main red pigment was peonidin-3-*O*-glucoside in the juice. The anthocyanin patterns of juices were much simpler compared to skins. In the berries of ‘Royalty’ which is a *Vitis rupestris* hybrid, anthocyanins in diglucoside forms were found at high concentrations. Surprisingly, two *Vitis vinifera* varieties, ‘Muscat Bouschet’ and ‘Kurucvér’, also contained malvidin-3,5-*O*-diglucosides of a significant amount. The anthocyanins patterns were found to be a useful tool to study the relationship between the cultivars. Our results help to choose the appropriate teinturier varieties with the desired anthocyanins for winemaking purposes or food coloring.

## Figures and Tables

**Table 1:** Mass spectrometer parameters for data acquisition.

Ion Source	HESI
Spray voltage	+/− 3 keV
Capillary temperature	350 °C
Probe heater temperature	300 °C
Flow rate of sheath gas (N_2_)	30 (a.u.)
Flow rate of auxiliary gas (N_2_)	10 (a.u.)
Resolution of Full Scan	35,000
Resolution of All Ion Fragmentation	17,500
S-Lens RF Level:	50%
Collision gas/energy	N_2_/30 eV

**Table 2:** Identified anthocyanins with the corresponding retention times (min) and m/z values (amu) obtained by electrospray ionization. Values in parentheses refer to ion abundance ratio.

No.	RT (min)	Compound	[M + H]^+^ (amu)	Positive Fragment Ions	[M − H]^−^ (amu)	Negative Fragment Ions
1	5.73 ± 0.03	Delphinidin-3-*O*-glucoside	465.1022	303.0495 (100), 304.0527 (16)	463.0889	300.0279 (100), 301.0344 (54)
2	7.07 ± 0.03	Petunidin-3,5-di-*O*-glucoside	641.1701	317.0650 (100), 479.1170 (17), 318.0681 (16)	639.1564	477.1032 (100), 315.0517 (67), 355.0669 (43)
3	7.26 ± 0.04	Cyanidin-3-*O*-glucoside	449.1071	287.0544 (100), 288.0577 (17)	447.0934	284.0327 (100), 285.0396 (87)
4	8.65 ± 0.03	Peonidin-3,5-di-*O*-glucoside	625.1760	301.0703 (100), 302.0736 (18), 463.1230 (18)	623.1627	479.1203 (100), 299.0564 (88), 461.1096 (84), 317.0668 (70)
5	8.90 ± 0.05	Pelargonidin-3-*O*-glucoside	433.1123	271.0591 (100), 272.0618 (17)	431.0982	269.0448 (100), 268.0378 (81), 270.0482 (16), 431.0982 (12)
6	9.67 ± 0.04	Petunidin-3-*O*-glucoside	479.1182	317.0652 (100), 318.0685 (18)	477.1043	314.0437 (100), 315.0508 (87), 300.0273 (21), 316.0542 (16)
7	10.51 ± 0.05	Malvidin-3,5-di-*O*-glucoside	655.1864	331.0806 (100), 332.0838 (19), 493.1335 (18)	653.1729	509.1306 (100), 329.0668 (60), 347.0775 (59), 491.1198 (54)
8	11.51 ± 0.04	Peonidin-3-*O*-glucoside	463.1230	301.0702 (100), 302.0736 (17), 349.1827 (12)	461.1094	299.0560 (100), 298.0486 (66), 284.0326 (21), 300.0602 (17)
9	13.94 ± 0.05	Malvidin-3-*O*-glucoside	493.1337	331.0807 (100), 332.0839 (19)	491.1200	329.0664 (100), 328.0592 (63), 314.0430 (19), 330.0698 (18)
10	16.08 ± 0.04	Delphinidin-3-*O*-(6-O-acetyl-glucoside)	507.1132	303.0497 (100), 304.0530 (17)	505.0994	300.0277 (100), 301.0346 (60)
11	18.70 ± 0.05	Malvidin-3-*O*-(6-O-acetyl-glucoside)-5-*O*-glucoside	697.1964	331.0810 (100), 349.1829 (59), 535.1443 (34), 332.0844 (19)	695.1840	509.1310 (100), 347.0779 (54), 329.0668 (49), 491.1180 (45)
12	21.42 ± 0.05	Petunidin-3-*O*-(6-O-acetyl-glucoside)	521.1287	437.2350 (100), 317.0650 (88), 437.2350 (19), 318.0683 (15)	519.1146	314.0436 (100), 315.0509 (96), 316.0542 (18)
13	24.22 ± 0.06	Peonidin-3-*O*-(6-O-acetyl-glucoside)	505.1333	301.0700 (100), 302.0732 (17), 525.2872 (12)	503.1197	299.0557 (100), 298.0483 (67), 300.0592 (17), 284.0323 (13)
14	25.68 ± 0.06	Delphinidin-3-*O*-(6-O-p-coumaroyl-glucoside)	611.1389	303.0496 (100), 525.2875 (21), 304.0529 (17)	609.1257	300.0278 (100), 301.0340 (41), 609.1265 (14)
15	26.69 ± 0.06	Malvidin-3-*O*-(6-O-acetyl-glucoside)	535.1444	331.0807 (100), 525.2877 (28), 332.0841 (18)	533.1306	329.0667 (100), 328.0592 (62), 330.0700 (18)
16	28.44 ± 0.08	Cyanidin-3-*O*-(6-O-p-coumaroyl-glucoside)	595.1440	287.0544 (100), 301.0695 (33)	593.1306	284.0327 (100), 285.0399 (73), 299.0564 (44)
17	30.05 ± 0.08	Malvidin-3-*O*-(6-O-coumaroyl-glucoside)-5-*O*-glucoside	801.2231	331.0808 (100), 639.1703 (66), 525.2877 (46), 640.1735 (25)	799.2097	509.1307 (100), 491.1201 (45), 637.1574 (39), 329.0670 (27)
18	30.29 ± 0.05	Malvidin-3-*O*-(6-O-cis-p-coumaroyl-glucoside)	639.1700	331.0807 (100), 332.0829 (17)	637.1567	329.0670 (100), 328.0583 (35), 330.0700 (18)
19	30.85 ± 0.06	Petunidin-3-*O*-(6-O-trans-p-coumaroyl-glucoside)	625.1548	317.0651 (100), 318.0684 (17), 613.3401 (12)	623.1414	314.0437 (100), 315.0506 (80), 623.1417 (15), 316.0543 (13)
20	33.50 ± 0.06	Peonidin-3-*O*-(6-O-trans-p-coumaroyl-glucoside)	609.1592	301.0698 (100), 302.0730 (18)	607.1461	299.0559 (100), 298.0484 (34), 300.0593 (17)
21	35.30 ± 0.06	Malvidin-3-*O*-(6-O-trans-p-coumaroyl-glucoside)	639.1700	331.0806 (100), 332.0838 (19)	637.1572	329.0668 (100), 328.0592 (32), 330.0702 (18)

**Table 3:** Level of the main anthocyanins of various teinturier grapevine cultivars.

	Dph-glc		Cyd-glc		Plg-glc		Ptd-glc		Mvd-3,5-diglc		Pnd-glc		Mvd-glc	
Cultivars	Skin	Juice	Skin	Juice	Skin	Juice	Skin	Juice	Skin	Juice	Skin	Juice	Skin	Juice
Alicante Bouschet	451 ± 42	trace	132 ± 3	1.7 ± 0.1	trace	trace	781 ± 40	Nd	nd	trace	2790 ± 60	80.8 ± 3.3	10,005 ± 214	22.8 ± 0.3
Bíborkadarka	768 ± 110	trace	207 ± 25	2.5 ± 0.4	trace	2.2 ± 0.1	1218 ± 181	Nd	nd	trace	2964 ± 317	90.4 ± 4.6	13,505 ± 1867	32.0 ± 1.6
Cabernet Mitos	1817 ± 68	trace	365 ± 3	41.7 ± 1.8	trace	trace	2316 ± 75	11.2±0.6	nd	trace	3398 ± 227	381.6 ± 24.9	13,665 ± 1580	320.3 ± 6.3
Carignan Bouchset	144 ± 8	trace	trace	4.5 ± 0.2	nd	trace	284 ± 7	trace	nd	nd	631 ± 13	118.0 ± 3.7	5987 ± 25	47.0 ± 2.3
Gamay Fréaux	949 ± 21	trace	230 ± 1	5.5 ± 0.4	trace	trace	1537 ± 82	trace	nd	trace	2755 ± 37	153.8 ± 6.8	15,045 ± 748	79.9 ± 3.4
Grand Noir	170 ± 10	trace	78 ± 13	1.0 ± 0.1	trace	2.4 ± 0.2	306 ± 10	Nd	nd	trace	3468 ± 395	100.9 ± 1.3	3894 ± 219	30.7 ± 0.4
Kármin	363 ± 30	trace	trace	1.8 ± 0.0	nd	1.1 ± 0.1	532 ± 49	nd	nd	trace	495 ± 48	71.4 ± 2.5	6281 ± 634	28.0 ± 1.1
Kurucvér	7933 ± 35	3.4 ± 0.2	738 ± 37	21.0 ± 2.8	trace	4.1 ± 0.1	4993 ± 132	6.1 ± 0.2	2168 ± 142	trace	927 ± 52	42.4 ± 1.3	15,201 ± 311	85.7 ± 2.8
Muscat Bouschet	408 ± 29	trace	115 ± 2	1.7 ± 0.2	trace	1.2 ± 0.1	631 ± 10	Nd	543 ± 10	trace	2263 ± 36	74.7 ± 2.6	6991 ± 40	16.2 ± 0.7
Oeillade Bouschet	7032 ± 237	2.9 ± 0.2	1054 ± 87	18.8 ± 0.7	trace	trace	5893 ± 240	6. 2 ± 0.9	nd	trace	2772 ± 230	143.3 ± 2.1	19,102 ± 901	93.4 ± 2.7
Palas	1700 ± 43	trace	410 ± 14	19.1 ± 0.7	trace	trace	2301 ± 24	6.4 ± 0.9	nd	trace	3237 ± 13	226.8 ± 9.1	11,454 ± 145	210.4 ± 7.9
Petit Bouschet	211 ± 24	trace	118 ± 6	2.9 ± 0.2	trace	1.6 ± 0.1	411 ± 38	nd	nd	nd	3959 ± 130	78.9 ± 3.1	5860 ± 65	13.7 ± 0.3
Royalty	611 ± 6	3.4 ± 0.7	trace	18.4 ± 0.5	nd	nd	795 ± 120	7.6 ± 0.3	15,264 ± 271	349.7 ± 6.9	337 ± 26	78.3 ± 0.9	2207 ± 176	51.6 ± 1.0
Teinturier	327 ± 18	trace	100 ± 13	2.3 ± 0.1	nd	trace	500 ± 25	nd	nd	nd	1402 ± 25	91.6 ± 3.1	5590 ± 103	15.7 ± 0.2
Turán	693.5 ± 52.4	5.0 ± 0.3	178 ± 15	14.4 ± 1.0	nd	1.8 ± 0.1	1065 ± 43	7.3 ± 0.7	nd	trace	1263 ± 52	106.6 ± 5.4	6633 ± 229	82.3 ± 4.0

Data are expressed in μg/g FW and mg/L for berry skin and juice, respectively. Mean values ± standard deviation; trace: trace amount of the compound was detected; nd: not detected. Dph-glc: Delphinidin-3-*O*-glucoside, Cyd-glc: Cyanidin-3-*O*-glucoside, Plg-glc: Pelargonidin-3-*O*-glucoside, Ptd-glc: Petunidin-3-*O*-glucoside, Mvd-3,5-diglc: Malvidin-3,5-di-*O*-glucoside, Pnd-glc: Peonidin-3-*O*-glucoside, Mvd-glc: Malvidin-3-*O*-glucoside.

**Table 4:**
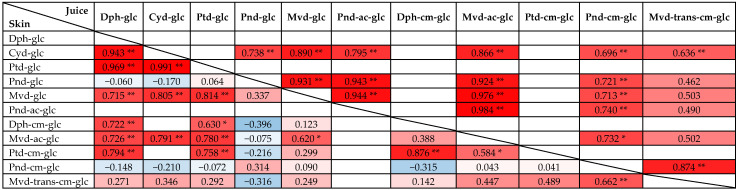
Pearson’s correlation coefficients between individual anthocyanins in juice and skin samples (n ≥ 10). The positive and negative correlations are displayed by red and blue colors, respectively. The color intensity is proportional to the correlation coefficients. White cells indicate when n < 10.

** Correlation is significant at the 0.01 level (2-tailed); * Correlation is significant at the 0.05 level (2-tailed).

## Data Availability

The unpublished data which may provide additional information in order to understand the present research are accessible from the corresponding author.

## Supplementary Materials

The following supporting information can be downloaded at: https://www.mdpi.com/article/10.3390/foods11223668/s1, Figure S1: Pedigrees of the studied teinturier grapevine varieties. Variety numbers in *Vitis* International Variety Catalogue (*V*IVC) are given in brackets. Varieties used in this study are marked with a red frame. If the pedigree is confirmed by SSR markers [*V*IVC, www.vivc.de], the cultivar name is highlighted with blue color. SSR markers did not confirm that ‘Bíborkadarka’ is the progeny of ‘Muscat Bouschet’; Figure S2: Dendrograms of grape (a) skin and (b) juice samples based on the similarities in their anthocyanin composition; Table S1: Analyzed grapevine accessions, genotypic data of 9 SSR loci and allele status at the berry color locus; Table S2. Berry weight and different parameters of juice samples obtained by pressing of pealed berry flesh; Table S3. Level of individual anthocyanins in berry skin samples of various teinturier grapevine cultivars; Table S4. Level of individual anthocyanins in berry juice samples of various teinturier grapevine cultivars.
