# Supplementary material for: Comparative Study on Grape Berry Anthocyanins of Various Teinturier Varieties
Source: Foods. 2022 Nov 16;11(22):3668. doi: 10.3390/foods11223668 (PMC9689461; doi:10.3390/foods11223668)
Supplement: Supplementary file 1 [file foods-11-03668-s001.zip › foods-2007770-supplementary.pdf]

Supplementary Material

Comparative study on grape berry anthocyanins of various teinturier varieties

László Kőrösi[1,*], Szilárd Molnár[1], Péter Teszlák[1], Ágnes Dörnyei[2], Erika Maul[3], Reinhard Töpfer[3], Tamás Marosvölgyi[4], Éva Szabó[5], Franco Röckel[3]

[1] Research Institute for Viticulture and Oenology, University of Pécs, 7634 Pécs, Hungary.
[2] Department of Analytical and Environmental Chemistry, Faculty of Sciences, University of Pécs, 7624 Pécs, Hungary.
[3] Julius Kühn Institute (JKI) - Federal Research Centre for Cultivated Plants, Institute for Grapevine Breeding Geilweilerhof, Siebeldingen, 76833, Germany.
[4] Institute of Bioanalysis, Medical School, University of Pécs, 7624 Pécs, Hungary.
[5] Department of Biochemistry and Medical Chemistry, Medical School, University of Pécs, 7624 Pécs, Hungary.

*Correspondence: korosi.laszlo@pte.hu

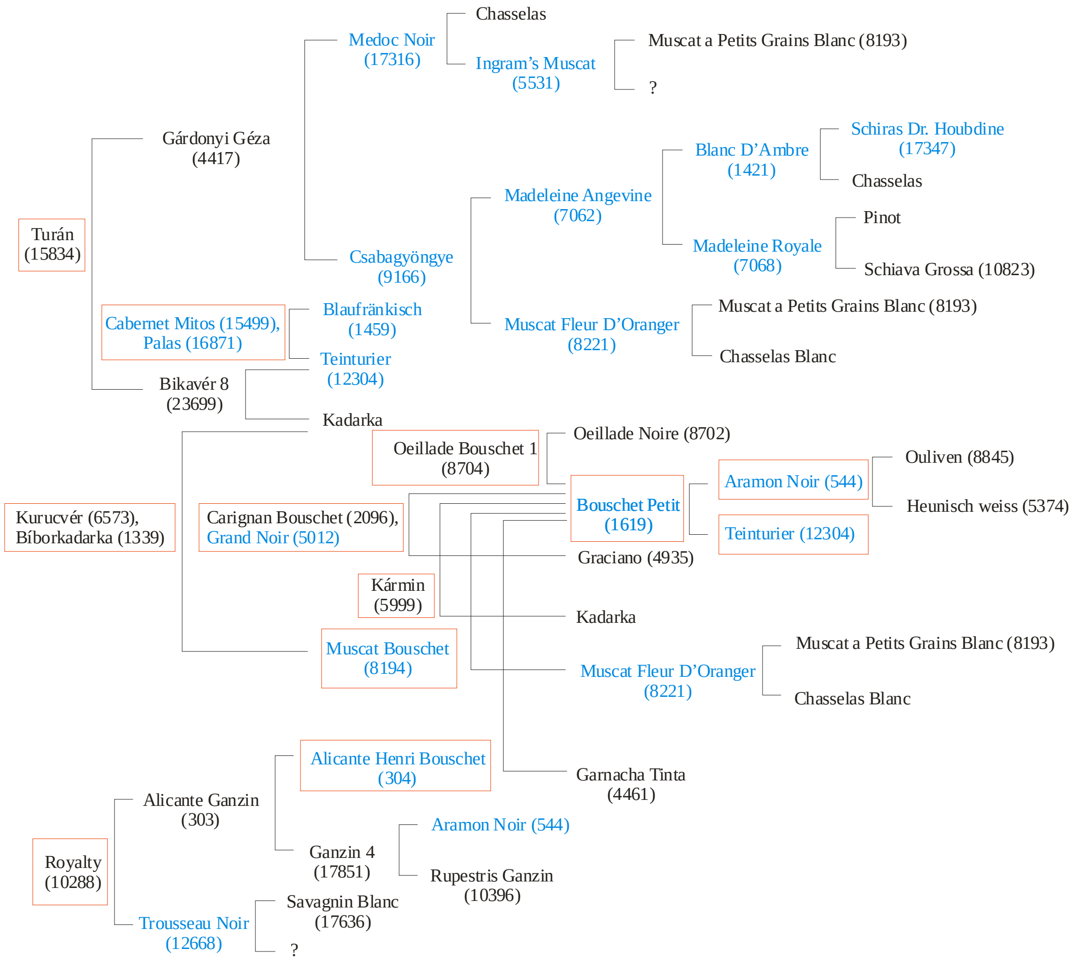

Figure S1. Pedigrees of the studied teinturier grapevine varieties. Variety numbers in *Vitis* International Variety Catalogue (*V*IVC) are given in brackets. Varieties used in this study are marked with a red frame. If the pedigree is confirmed by SSR markers [*V*IVC, www.vivc.de], the cultivar name is highlighted with blue color. SSR markers did not confirm that 'Bíborkadarka' is the progeny of 'Muscat Bouschet'.

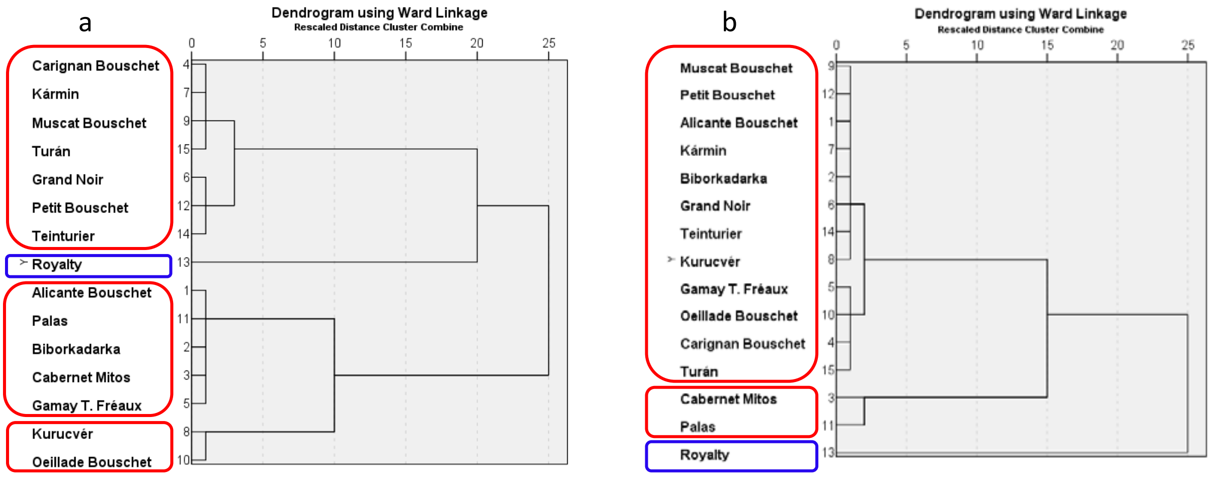

Figure S2. Dendrograms of grape (a) skin and (b) juice samples based on the similarities in their anthocyanin composition.

Table S1. Analyzed grapevine accessions, genotypic data of 9 SSR loci and allele status at the berry color locus.

Prime name (Official Variety Name)	Accession Name (Name in Repository)	Variety number *V*IVC	*MybA1a* white	*MybA1c* red	*MybA1t* repeat number	VVMD27		VVS2		VVMD7		VVMD5		VrZAG62		VrZAG79		VVMD28		VVMD32		VVMD25	
Alicante Henri Bouschet	Alicante Bouschet	304	-	x	2	182	195	133	145	239	243	228	240	188	188	243	257	244	260	250	272	241	241
Biborkadarka	Bíborkadarka	1339	-	x	2	195	195	133	145	239	255	228	228	188	204	251	257	244	260	272	272	239	241
Cabernet Mitos	Cabernet Mitos	15499	x	-	5	180	195	137	143	239	239	228	240	194	194	251	255	228	246	250	272	239	249
Carignan Bouschet	Carignan Bouschet	2096	x	-	2	182	195	137	143	239	243	236	240	188	196	243	257	228	260	250	250	249	255
Gamay Teinturier Freaux	Gamay Fréaux	4382	x	x	-	182	190	133	137	239	249	236	240	194	204	243	245	218	246	240	272	239	239
Grand Noir	Grand Noir	5012	-	x	2	182	184	139	151	239	243	228	236	188	188	243	259	236	244	240	250	249	271
Karmin	Kármin	5999	-	x	2	182	186	133	133	239	255	228	240	196	204	243	251	228	260	250	272	239	241
Kurucver	Kurucvér	6573	-	x	2	180	195	133	133	239	255	228	240	196	204	249	255	228	260	250	272	239	249
Muscat Bouschet	Muscat Bouschet	8194	x	-	2	180	190	133	151	239	247	238	240	196	204	245	255	246	260	240	250	249	249
Oeillade Bouschet 1	Oeillade Bouschet	8704	-	x	2	182	182	143	143	239	239	228	228	186	194	259	259	228	258	250	250	255	255
Palas	Palas	16871	x	-	5	180	195	137	143	239	239	240	242	194	204	251	255	228	246	272	272	239	249
Bouschet Petit	Petit Bouschet	1619	-	x	2	182	190	133	151	239	243	236	240	188	196	243	245	236	260	250	272	241	249
Royalty	Kék Royalti	10288	-	x	2	176	195	145	151	249	257	236	240	188	198	247	257	228	248	250	256	241	249
Teinturier	Teinturier Femelle	12304	x	x	3	180	190	137	151	239	243	240	240	188	194	245	255	228	236	272	272	239	249
Turan	Turán	15834	-	x	2	180	180	133	133	239	249	228	238	186	194	255	255	218	260	240	272	239	241

Table S2. Berry weight and different parameters of juice samples obtained by pressing of pealed berry flesh.

Cultivar	Weight (g/berry)	pH	TSS (°Brix)	tartaric acid (g/L)	malic acid (g/L)	citric acid (g/L)
Alicante Bouschet	2.35	3.4	17.3	2.30	5.67	0.26
Bíborkadarka	2.92	3.8	17.4	1.96	3.31	0.18
Cabernet Mitos	1.54	4.0	23.0	4.88	4.94	0.40
Carignan Bouschet	1.54	3.1	16.2	3.63	2.60	0.22
Gamay Fréaux	1.81	3.4	20.6	3.62	3.87	0.18
Grand Noir	2.95	3.3	16.2	2.84	2.54	0.20
Kármin	2.95	3.1	17.8	2.43	4.84	0.23
Kurucvér	1.52	4.0	18.5	2.31	3.15	0.16
Muscat Bouschet	1.01	4.2	21.5	2.56	3.47	0.22
Oeillade Bouschet	2.08	3.0	17.0	3.62	3.81	0.22
Palas	2.21	4.1	21.9	2.94	5.32	0.34
Petit Bouschet	1.51	3.4	16.8	2.27	3.52	0.21
Royalty	2.39	3.4	18.5	3.52	2.69	0.16
Teinturier	1.67	3.5	17.5	2.54	4.14	0.25
Turán	1.64	4.3	23.4	3.87	2.32	0.22

Table S3. Level of individual anthocyanins in berry skin samples of various teinturier grapevine cultivars.

Cultivars	Ptd-3,5-diglc	Pnd-3,5-diglc	Dph-ac-glc	Mvd-3-ac-glc-5-glc	Ptd-ac-glc	Pnd-ac-glc	Dph-cm-glc	Mvd-ac-glc	Cyd-cm-glc	Mvd-3-cm-glc-5-glc	Mvd-cis-cm-glc	Ptd-cm-glc	Pnd-cm-glc	Mvd-trans-cm-glc
Alicante Bouschet	nd	nd	trace	nd	nd	90.9±0.1	134.1±23.7	512.0±28.1	trace	trace	trace	212.3±9.1	835.4±44.4	3589.4±354.7
Bíborkadarka	nd	trace	trace	nd	trace	88.7±9.0	267.3±43.1	644.6±86.2	92.2±8.9	trace	170.6±59.1	357.1±51.4	903.4±95.0	5078.0±631.2
Cabernet Mitos	nd	trace	418.4±25.7	trace	475.9±35.9	530.9±26.2	428.9±26.3	3002.9±316.8	trace	trace	trace	466.5±12.0	752.4±1.6	3965.1±209.0
Carignan Bouschet	nd	nd	nd	nd	nd	87.1±1.0	99.3±0.7	1198.8±47.3	nd	107.1±4.5	82.2±6.6	191.1±1.5	375.0±3.5	4809.5±252.9
Gamay Fréaux	nd	nd	nd	nd	nd	trace	trace	313.5±18.1	trace	trace	144.6±2.9	88.2±3.6	193.8±15.8	1123.3±50.4
Grand Noir	nd	nd	trace	nd	trace	trace	trace	165.2±3.3	trace	trace	trace	trace	414.2±10.6	899.3±10.1
Kármin	nd	trace	trace	nd	86.5±6.2	trace	146.3±13.6	1166.6±149.5	tace	trace	trace	192.0±11.6	206.7±11.9	3029.1±195.2
Kurucvér	nd	313.4±105.6	1513.1±108.6	208.7±17.3	947.5±32.7	82.1±11.0	2511.7±248.1	2502.3±183.0	248.7±25.2	254.0±19.6	trace	1240.7±13trace	192.6±19.6	3803.8±452.0
Muscat Bouschet	nd	147.2±18.8	trace	nd	trace	trace	231.4±8.4	312.3±15.8	trace	250.9±19.0	119.9±32.5	305.0±6.4	980.9±6.1	3993.9±13.4
Oeillade Bouschet	nd	nd	927.1±39.4	86.6±0.3	843.7±48.9	200.9±0.4	1637.7±99.3	2751.9±126.4	279.1±5.9	trace	trace	1355.6±87.9	613.4±24.0	5444.9±387.5
Palas	nd	nd	286.6±16.7	trace	360.9±11.4	342.2±30.3	233.4±0.9	1665.4±126.8	trace	trace	113.3±6.6	247.3±2.4	394.7±6.6	1513.4±37.2
Petit Bouschet	nd	trace	trace	nd	nd	trace	trace	155.5±24.9	trace	trace	64.0±1.0	trace	552.9±5.5	1166.4±72.6
Royalty	820.5±0.7	1754.9±278.7	92.6±4.1	906.1±38.8	93.6±11.2	trace	2171.8±17.1	460.2±109.7	491.1±45.4	7070.9±324.6	nd	848.2±6.3	176.6±12.2	2951.0±11.0
Teinturier	nd	nd	trace	nd	72.1±4.2	140.8±2.6	trace	862.1±5.4	nd	trace	94.0±4.0	trace	192.6±1.3	1190.2±11.8
Turán	nd	nd	96.7±1.7	nd	157.4±8.8	210.2±4.4	697.8±64.9	1474.7±49.2	237.4±22.6	118.6±17.2	154.1±6.2	922.1±91.3	1265.0±110.8	7240.1±715.4

Data are expressed in malvidin-3-*O*-glucoside equivalent µg/g FW as mean values ± standard deviation; nd: not detected. Ptd-3,5-diglc: Petunidin-3,5-di-*O*-glucoside, Pnd-3,5-diglc: Peonidin-3,5-di-*O*-glucoside, Dph-ac-glc: Delphinidin-3-*O*-(6-*O*-acetyl-glucoside), Mvd-3-ac-glc-5-glc: Malvidin-3-*O*-(6-*O*-acetyl-glucoside)-5-*O*-glucoside, Ptd-ac-glc: Petunidin-3-*O*-(6-*O*-acetyl-glucoside), Pnd-ac-glc: Peonidin-3-*O*-(6-O-acetyl-glucoside), Dph-cm-glc: Delphinidin-3-*O*-(6-*O*-p-coumaroyl-glucoside), Mvd-ac-glc: Malvidin-3-*O*-(6-*O*-acetyl-glucoside), Cyd-cm-glc: Cyanidin-3-*O*-(6-*O*-p-coumaroyl-glucoside), Mvd-cm-glc-5-glc: Malvidin-3-*O*-(6-*O*-coumaroyl-glucoside)-5-*O*-glucoside, Mvd-3-cis-cm-glc: Malvidin-3-*O*-(6-*O*-cis-p-coumaroyl-glucoside), Ptd-cm-glc: Petunidin-3-*O*-(6-*O*-trans-p-coumaroyl-glucoside), Pnd-cm-glc: Peonidin-3-*O*-(6-*O*-trans-p-coumaroyl-glucoside), Mvd-trans-cm-glc: Malvidin-3-*O*-(6-*O*-trans-p-coumaroyl-glucoside).

Table S4. Level of individual anthocyanins in berry juice samples of various teinturier grapevine cultivars.

Cultivars	Ptd-3,5-diglc	Pnd-3,5-diglc	Dph-ac-glc	Mvd-3-ac-glc-5-glc	Ptd-ac-glc	Pnd-ac-glc	Dph-cm-glc	Mvd-ac-glc	Cyd-cm-glc	Mvd-3-cm-glc-5-glc	Mvd-cis-cm-glc	Ptd-cm-glc	Pnd-cm-glc	Mvd-trans-cm-glc
Alicante Bouschet	nd	nd	nd	nd	nd	3.1±0.0	nd	trace	trace	nd	nd	nd	5.2±0.4	1.3±0.1
Bíborkadarka	nd	nd	nd	trace	nd	1.9±0.1	nd	1.0±0.1	trace	nd	trace	nd	6.2±0.3	2.4±0.2
Cabernet Mitos	nd	nd	trace	4.6±0.2	trace	65.4±2.2	nd	69.9±1.3	6.5±0.5	nd	3.6±0.2	trace	19.7±0.5	15.2±0.4
Carignan Bouschet	nd	nd	nd	nd	nd	6.8±0.1	nd	4.2±0.1	trace	nd	trace	nd	6.3±0.5	4.4±0.2
Gamay Fréaux	nd	nd	nd	nd	nd	1.3±0.0	nd	1.2±0.0	trace	nd	trace	nd	1.8±0.2	1.2±0.1
Grand Noir	nd	nd	nd	nd	nd	1.2±0.0	nd	trace	trace	nd	nd	nd	1.6±0.2	trace
Kármin	nd	nd	nd	nd	nd	4.5±0.2	nd	2.7±0.1	nd	nd	nd	nd	3.8±0.3	2.0±0.1
Kurucvér	nd	nd	trace	1.9±0.2	trace	3.1±0.1	nd	10.8±0.4	trace	nd	trace	trace	1.4±0.1	3.3±0.4
Muscat Bouschet	nd	nd	nd	nd	nd	1.1±0.0	nd	trace	trace	nd	trace	nd	2.0±0.1	trace
Oeillade Bouschet	nd	nd	trace	trace	trace	5.8±0.1	nd	11.2±0.1	trace	nd	trace	trace	5.7±0.3	7.1±0.7
Palas	nd	nd	trace	1.9±0.1	trace	34.2±0.4	nd	34.3±1.5	3.9±0.3	nd	2.3±0.2	nd	5.1±0.3	3.5±0.3
Petit Bouschet	nd	nd	nd	nd	nd	1.6±0.1	nd	trace	trace	nd	nd	nd	2.7±0.2	trace
Royalty	nd	434.0±3.7	trace	10.8±0.1	trace	2.8±0.1	trace	3.2±0.1	43.9±3.1	42.1±3.0	nd	trace	4.7±0.5	6.8±0.6
Teinturier	nd	nd	trace	nd	nd	7.9±0.1	nd	1.9±0.1	trace	nd	nd	nd	1.3±0.1	trace
Turán	nd	nd	trace	trace	trace	7.2±0.4	trace	9.1±0.4	2.4±0.2	nd	trace	1.5±0.1	14.2±0.9	18.1±1.7

Data are expressed in malvidin-3-*O*-glucoside equivalent mg/L as mean values ± standard deviation; nd: not detected. Ptd-3,5-diglc: Petunidin-3,5-di-*O*-glucoside, Pnd-3,5-diglc: Peonidin-3,5-di-*O*-glucoside, Dph-ac-glc: Delphinidin-3-*O*-(6-*O*-acetyl-glucoside), Mvd-3-ac-glc-5-glc: Malvidin-3-*O*-(6-*O*-acetyl-glucoside)-5-*O*-glucoside, Ptd-ac-glc: Petunidin-3-*O*-(6-*O*-acetyl-glucoside), Pnd-ac-glc: Peonidin-3-*O*-(6-O-acetyl-glucoside), Dph-cm-glc: Delphinidin-3-*O*-(6-*O*-p-coumaroyl-glucoside), Mvd-ac-glc: Malvidin-3-*O*-(6-*O*-acetyl-glucoside), Cyd-cm-glc: Cyanidin-3-*O*-(6-*O*-p-coumaroyl-glucoside), Mvd-3-cm-glc-5-glc: Malvidin-3-*O*-(6-*O*-coumaroyl-glucoside)-5-*O*-glucoside, Mvd-cis-cm-glc: Malvidin-3-*O*-(6-*O*-cis-p-coumaroyl-glucoside), Ptd-cm-glc: Petunidin-3-*O*-(6-*O*-trans-p-coumaroyl-glucoside), Pnd-cm-glc: Peonidin-3-*O*-(6-*O*-trans-p-coumaroyl-glucoside), Mvd-trans-cm-glc: Malvidin-3-*O*-(6-*O*-trans-p-coumaroyl-glucoside)
